# Super Soft All-Ethylene Oxide Polymer Electrolyte for Safe All-Solid Lithium Batteries

**DOI:** 10.1038/srep19892

**Published:** 2016-01-21

**Authors:** Luca Porcarelli, Claudio Gerbaldi, Federico Bella, Jijeesh Ravi Nair

**Affiliations:** 1 GAME Lab, CHENERGY Group, Department of Applied Science and Technology – DISAT, Politecnico di Torino, Corso Duca degli Abruzzi 24, 10129-Torino, Italy

## Abstract

Here we demonstrate that by regulating the mobility of classic −EO− based backbones, an innovative polymer electrolyte system can be architectured. This polymer electrolyte allows the construction of all solid lithium-based polymer cells having outstanding cycling behaviour in terms of rate capability and stability over a wide range of operating temperatures. Polymer electrolytes are obtained by UV-induced (co)polymerization, which promotes an effective interlinking between the polyethylene oxide (PEO) chains plasticized by tetraglyme at various lithium salt concentrations. The polymer networks exhibit sterling mechanical robustness, high flexibility, homogeneous and highly amorphous characteristics. Ambient temperature ionic conductivity values exceeding 0.1 mS cm^−1^ are obtained, along with a wide electrochemical stability window (>5 V *vs.* Li/Li^+^), excellent lithium ion transference number (>0.6) as well as interfacial stability. Moreover, the efficacious resistance to lithium dendrite nucleation and growth postulates the implementation of these polymer electrolytes in next generation of all-solid Li-metal batteries working at ambient conditions.

Present energy storage and production devices are based on combustible organic solvents that carry the risks of leakage and related fire hazards. This forces the manufacturer to enclose battery components in heavier and peculiar packaging structures to meet the stringent safety requisites. Such heavy protective packaging reduces the overall amount of ready-to-use energy (energy density). An all-solid construction will certainly enhance the overall performance of any energy storage and conversion devices that can be thought of^1^.

An ideal ion conducting polymer electrolyte for ambient temperature energy storage has been the dream pursued by many researchers^2^,^3^,^4^. An enhanced safety may be achieved by complete replacement of organic carbonate-based liquid electrolytes, thus leading to facile and leak-free fabrication, flexibility, compactness, reduced weight and laminated structures^5^. Scientific community has been focusing on polymer systems containing –EO– moieties for an all-solid cell construction, and other polymers (e.g., PMMA, PVdF) in mostly hybrid/gel configurations^6^,^7^. It is well known that polyethylene oxide (PEO) crystallizes at about 55 °C^8^,^9^ and ionic as well as segmental mobility are limited to its molten (amorphous) state at elevated temperatures. This impedes its widespread application into the global market^10^. However, at elevated temperatures such polymers lose their dimensional stability being in molten state, which leads to non-homogeneity inside the cell^11^. This facilitates the diffusion of Li^+^ ions through localized favourable paths, increasing both concentration gradients and defects that may create temperature deflections and short-circuits. Attempts have been also made to reduce the crystallinity to retain the amorphicity by incorporating various ceramics/metal oxides (e.g., Al_2_O_3_, CeO_2_, ZrO_2_, TiO_2_)^12^, but the target remains unachieved so far. Another problem is the solvent-based preparation procedure, where complete and “concrete” solvent removal is a herculean task; indeed, in most cases the unavoidable traces of solvent persisting in the polymer matrix create various interfacial stability issues, even enabling thermal runaway reactions.

Four decades after the discovery of ionic conduction in polymer electrolytes by Fenton *et al.*^13^ and Armand *et al.*^14^, the topic remains red hot. Moreover, the all-solid-state Li-metal battery concept is still appealing due to assured high energy density^15^. A polymer electrolyte that is less reactive towards Li-metal and highly resistant to dendrite formation/penetration would potentially open up the possibility of Li-metal based accumulators to entry in the market. After decades of efforts, PEO matrix still struggles to meet the requirements of global market due to its low ambient temperature conductivity and inferior safety arising from non-uniform plating/stripping of Li^+^ ions, which often results in hazardous dendrite formation.

A compromise between the characteristics of an all-solid state^16^ and a gel-like^17^ polymer electrolyte might represent the vital knot that researchers need to accomplish the goal. In this direction, remarkable results are reported in literature with the addition of room temperature ionic liquids^18^ into thermoplastic^19^ or thermoset^20^ polymer matrix. However, the problems such as restricted Li^+^ ion diffusion, cost and rate capabilities become the hurdle towards the commercialisation of such materials. Thus, slowly and steadily the scientific community is moving towards the addition of noncarbonated high boiling, thermally stable organic solvents such as organic nitriles^21^, glymes^22^, etc. Moreover, in the case of thermoplastic polymers the separator size deformation with varying temperature is a tough issue, while thermosets are limited by unsuitable ionic conductivity and brittleness of the polymer matrix.

Glymes (various lengths) are well known for complexing with metal-ions through their multiple ether-like oxygen atoms^23^. When lithium salt is dissolved in glyme-based solvents, they show promising ionic conductivity and Li^+^ ion transport properties^24^,^25^. Due to the excellent properties imparted by glymes in the liquid electrolyte, recently they have received plenty of attention for next-gen systems beyond Li-ion, such as lithium sulphur^2^,^3^,^4^ and lithium air rechargeable batteries^26^,^27^,^28^.

Free radical photopolymerisation is a low cost, solvent-free and energy saving technique very well established for many applications in an easily implemented and versatile fashion^29^,^30^,^31^. Photopolymerisation can be suitably adapted to the preparation of polymer electrolytes due to its eco-friendliness, which is a key aspect that influences the fate of large-scale polymer electrolyte manufacturing^32^. Moreover, UV-induced reaction on multifunctional monomers permits rapid *in situ* generation of intimate electrode/electrolyte interfaces, which currently represent a major striking point to be fixed in the field of electrochemical devices^33^. Thus, the whole electrolyte preparation can be carried out in the absence of solvent.

Most of the systems referring to glyme-based electrolytes are either blended with thermoplastic materials or directly used in their liquid form. Little work^34^,^35^ has been devoted to study their possible implementation in a self-standing, softly cross-linked thermoplastic polymer matrix. In the present work, we use a system based on PEO and tetraglyme, and we directly cross-link it in one-pot along with the supporting lithium salt under UV irradiation to retain the solid-like nature and dimensional stability. By concurrent exploitation of photo-induced cross-linking and *in situ* functionalization procedures, kinetically driven inhibition of the PEO chains crystallization is readily achievable at ambient conditions, leading to polymer electrolytes that possess solid-like properties without hampering ionic mobility. They are prepared by mixing PEO as the polymer matrix, bis[2-(2-methoxyethoxy)ethyl]ether (tetraglyme, TEGDME) as the active plasticizer, lithium bistrifluoromethane sulfonimide (LiTFSI) as the source of Li^+^ ions and 4-methyl benzophenone (MBP) as the light-induced hydrogen abstraction mediator (photoinitiator). Under UV excitation, MBP abstracts an acidic proton from a methylene group and generates a free radical chain^36^,^37^. This free radical can combine with another free radical belonging to the same chain or other –EO– chains to interlink themselves. Tetraglyme also possesses methylene groups that can undergo hydrogen abstraction and following inter-radical reactions to form oligomers, or bond to the adjacent PEO chains. The final interlinked solid polymer electrolyte (ISPE) films are mechanically robust, highly flexible, homogeneous and largely amorphous. They also exhibit excellent properties in terms of compatibility with the lithium metal electrode and suppression of hazardous dendrite growth. The sum of these characteristics enlighten the striking prospects of the newly developed ISPE as electrolyte separators in both Li-ion and Li metal batteries conceived for high energy and/or power demanding applications, including hybrid vehicles and smart grid storage systems.

## Results and Discussions

### Physical-chemical characterization

**Figure 1:**
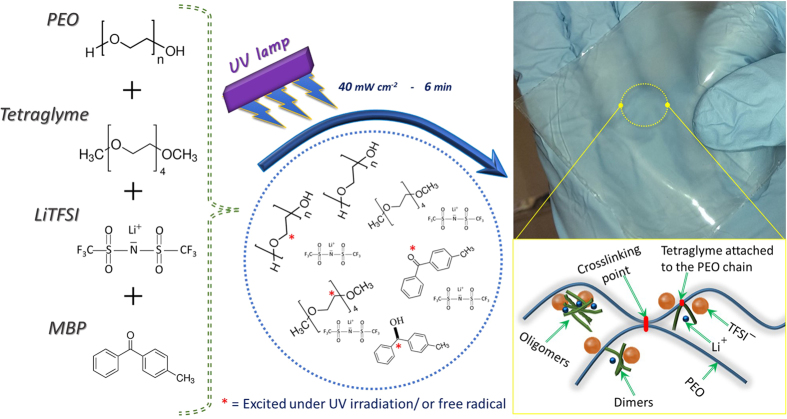
Sketched representation of ISPE preparation along with used materials, and plausible illustration (right bottom) of interconnected PEO chains with hypothesized branched clusters of tetraglyme oligomers; on the top right, the real aspect of a freshly prepared ISPE.

Figure 1 shows the materials used in the study along with the real aspect of ISPE formed after UV exposure (right top), which is almost transparent, tack free, flexible and easy to manage. On the right bottom side of Fig. 1, the possibility of interlinking of polymer chains is hypothesized, with the *in situ* oligomer formation and plausible grafting of TEGDME molecules onto the long PEO chains upon 6  min. of UV irradiation (intensity on the surface of the sample of 40 mW cm^−2^).

Before finalising to the formulations reported in this work, several tests were performed to understand the fundamental aspects of polymer electrolytes, to decide the quantity and type of photoinitiator, as well as the suitable molecular weight of PEO depending on the easiness in processing. MBP was selected as the photo-cross-linker with superior solubility and optimum cross-linking properties in quantities equal to 7.5 wt. % with respect to the total materials’ mass. Such studies are not included here being out of scope of the present discussion. The overall characteristics and composition of the different samples under study are listed in Table 1, including the glass transition temperature (*T*_g_) values and the gel content (insoluble fraction after cross-linking) against the corresponding –EO– to Li ratio.

**Figure 2:**
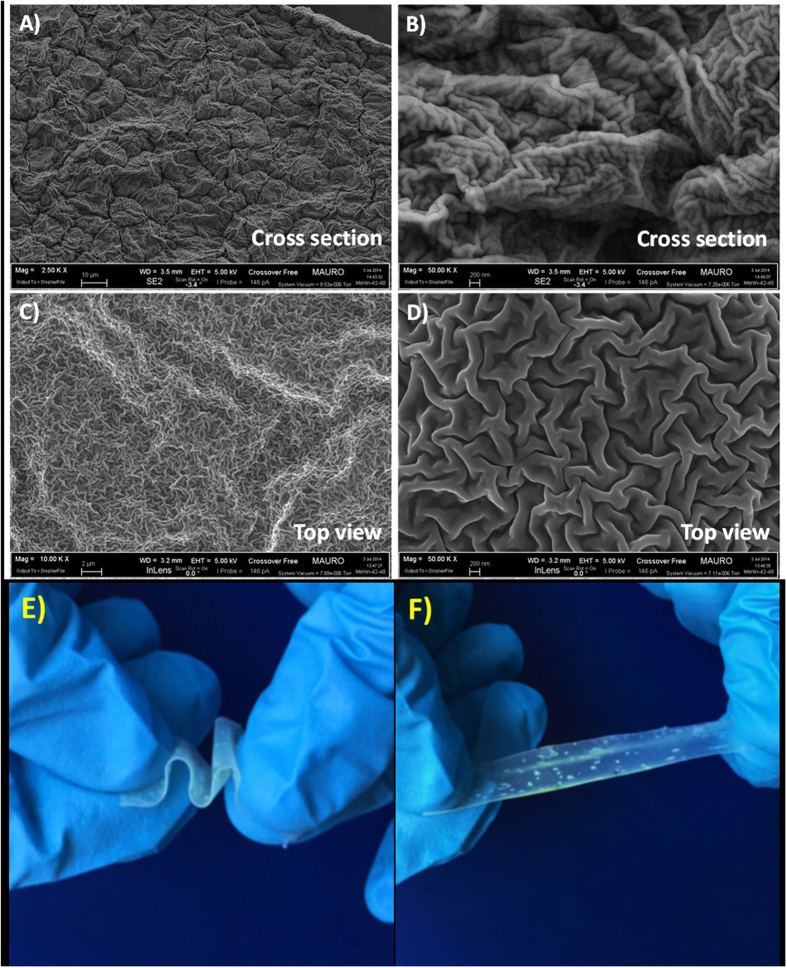
Micrographs showing the overall morphology of sample PTL-1: cross-section under secondary electron mode (**A,B**) and top view (**C,D**), at different magnifications; (**E,F**) shown the images of the sample PTL-1 (at 25 °C) under stretch and bend mode, demonstrating the mechanical integrity and excellent elasticity.

High-resolution FESEM analysis at 50K magnification (Fig. 2A–D) shows the characteristics of a soft, cross-linked polymer electrolyte (namely PTL-1) with rather high degree of amorphous nature. The micrographs are well in agreement with the results obtained by Schulze *et al.*, who used a one-pot synthetic strategy based on polymerization-induced phase separation to generate nanostructured polymer electrolytes that exhibited an unprecedented combination of high modulus and ionic conductivity^38^. In the present case, phase separation is not possible as the material is made of similar –EO– based backbones. Indeed, viscosity as well as polymerization induced aggregation and-to a certain extent - rearrangement of the PEO chains could be possible. The photopolymerisation was carried out after keeping the highly viscous reactive mixture under stressful conditions (90 °C, pressed at 20 bar) between two Mylar foils^39^. In particular, micrographs clearly evidence a bag-like polymer matrix that incorporates an extremely soft material. The surface roughness is very clearly visible in secondary electron mode. Moreover, the overall aspect and characteristics indicate the exceptional homogeneity of the sample, with no noticeable presence of pores or voids. Moreover, the images showed in Fig. 2(E,F) demonstrate that the obtained ISPE is stretchable and highly elastic (once the stress is released, it can go back to the previous shape). These results all together confirm that the proposed electrolyte is soft, weakly cross-linked, flexible, and shape retaining at ambient conditions.

**Figure 3:**
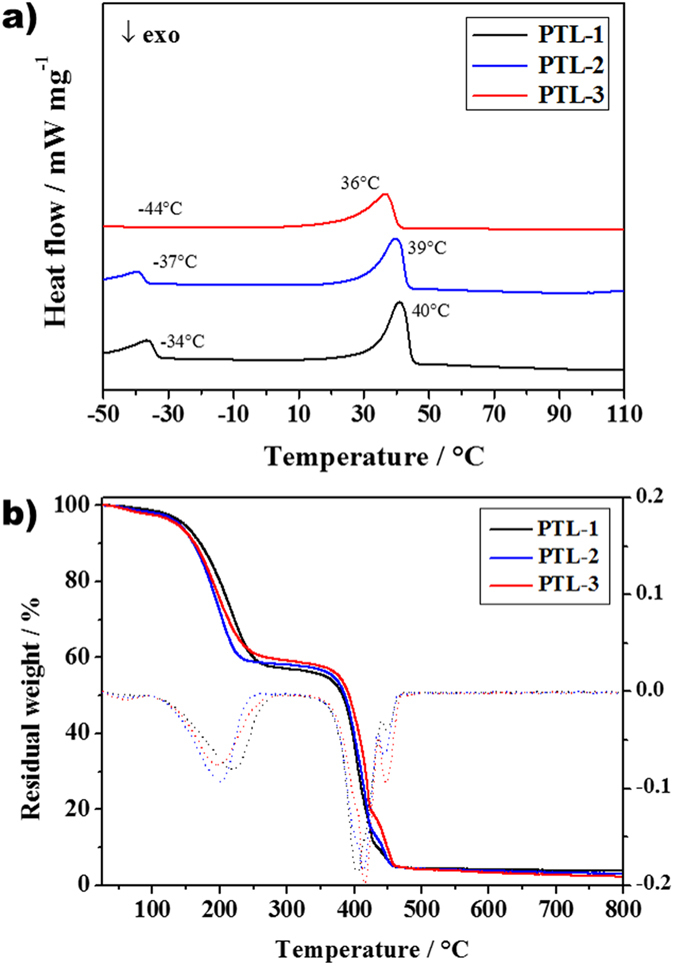
(**a**) Differential scanning calorimetry (DSC) curves of the ISPEs PTL-1 to PTL-3 that contains various amounts of LiTFSI salt. (**b**) Thermogravimetric analysis (TGA) of the same series of ISPEs along with related differential curves (dotted lines of same color code). Taking into account the experimental errors related to the measurement and the sample preparation, all weight losses are consistent with the polymer electrolyte compositions.

The differential scanning calorimetry (DSC) values are tabulated in Table 1, and the respective profiles are shown in Fig. 3A. Both the glass transition and the melting temperatures were determined for all the samples. *T*_g_ values vary between –34 and –44 °C. As expected, the amount of salt noticeably influences the *T*_g_ of the ISPEs prepared with 1:1 tetraglyme to PEO ratio. Generally, low *T*_g_ values account for a moderately enhanced segmental motion of the –EO– moieties in the polymer matrix which is facilitated by low crystallinity. Moreover, a noticeable change in the peak associated with the melting of the crystalline region is clearly evidenced: an increase in LiTFSI content reduces the intensity of the melting peak, which also results broadened. As noted in Table 1, an increase in salt content reduces the [EO]/[Li] ratio from 54:1–23:1. Thus, an increased amount of Li^+^ ions is available for the coordination with PEO chains, thus reducing the tendency of forming crystalline phases. Moreover, the cross-linking effect further reduces the mobility of the PEO main chains. Overall, a relationship is present between the phase transition temperature and the salt content: the higher is the salt content, the lower is the transition temperature. This is expected as for the typical coordination effect by lithium salt, which weakly decreases the *T*_g_^40^. Furthermore, ISPEs in all cases exhibit crystalline melting peaks near room temperature during the heating cycle of the DSC analysis. This may be attributed to low cross-linking density (7.7 × 10^3^ mol m^–3^ for PTL-1, calculated at 0 °C), which allows the long enough –EO– chains to rearrange and crystallize in the matrix^41^. It is worth noting that the melting points of these newly elaborated polymer electrolytes are well below the typical PEO-based electrolytes when tested by means of DSC^40^,^42^. If one calculates the number of MBP molecules per –EO– moieties, the latter exceeds 46 ± 3 per each photoinitiator molecule. This ratio is sufficient to enforce the rearrangement of the –EO– moieties to form weak crystalline phases. One cannot neglect the effect of tetraglyme in diluting the number of cross-linking per area, as some of the initiator molecules are actively involved in real-time oligomerization and eventual branching processes. Thus, the effective cross-linking would be lower than the theoretical calculations from mole ratios. Overall, the average cross-linking length obtained in the present work suggests that cross-linking is not sufficient to prevent the PEO crystallization. It can be hypothesized that a higher degree of cross-linking may not be also favoured, as it may induce the low molecular weight tetraglyme and/or its oligomers to squeeze out of the system during thermal stresses. Thus, we selected an optimum cross-linking, which could assure a good mechanical integrity along with good plasticizer retention (leak free).

The thermal gravimetric analysis (TGA) (Fig. 3b) shows three main weight losses for all the samples. The first one is associated to tetraglyme degradation/evaporation, the second one to PEO decomposition and the last one to lithium salt decomposition. The first dip before 100 °C indicates the loss of humidity that may be absorbed during the handling of the sample for testing. Taking into account the experimental errors related to the measurement and the sample preparation, the overall weight loss is consistent with the polymer electrolyte composition. According to the differential thermal analysis (shown in dotted lines), the weight loss occurs for all the samples above 190 °C, the major contributor being tetraglyme^41^. Higher amounts of LiTFSI reduces the thermal stability of the polymer matrix, which is clearly visible in the differential curves (shift of the peak from 218 to 195 °C). This could be the general trend observed for tetraglyme kind of plasticizers that show the tendency to decompose in reaction with fluorinated anions^22^. Thus, both DSC and TGA profiles show that no unusual phase changes or weight losses occur in the temperature range between 25 and 150 °C, which makes the material thermally stable and useful as a polymer electrolyte under standard operating conditions in real battery configuration.

Dynamic mechanical thermal analysis (DMTA) was performed on sample PTL-1 and the resulting profiles (both modulus and loss modulus) are shown in Figure S1(A,B). It clearly indicates that the material has low *T*g, which is in agreement with the DSC analysis. In addition, the calculated cross-linking density (7.7 × 10^3^ mol m^–3^, calculated at 0 °C) was found to be comparatively lower than other known systems^43^. This is an indication that the number of cross-linking points between the PEO chains is lower than expected, which allows the long enough –EO– chains to rearrange and crystallize in the matrix. Tensile tests were carried out on the PTL-1 sample according to ASTM Standard D638; the Young’s modulus was found to be 0.3 MPa and maximum force at break was found to be 1.5 MPa. The material can stretch (Figure S1C) very well under stress, as justified by the maximum strain (elongation) of around 17 mm before the membrane was broken.

The cross-linking effects and related properties were studied by an indirect method where the ISPE was subjected to dissolution in tetrahydrofuran (THF). The solubilized fraction was analysed using size exclusion chromatography/gel-permeation chromatography (SEC/GPC) to calculate the molecular weight of oligomers formed by the bonding between various tetraglyme molecules. We chose PTL-1 as the representative sample and performed the test to understand the molecular weight of the soluble fraction. It is worth noting that the results of insoluble fraction study are in agreement with the gel-content studies performed on similar samples. The soluble fraction in THF while testing showed a broad molecular weight distribution primary caused by the tetraglyme molecules. Molecules with various molecular weights ranging from 300 up to 41000 Da eluted from the column at different time intervals (Figure S2), which correspond to the higher homologous of tetraglyme such as dimers, trimers or oligomers. This is a direct indication that tetraglyme molecules are taking part in the polymerization reaction to form oligomers and remain active upon UV irradiation. In such a scenario, some branches that arise from the reactions between the radicals generated from –EO– of PEO and –EO– of tetraglyme might be formed. The oligomers formed by tetraglyme may not remain in the polymer matrix, which is clearly visible when the membrane was placed in the THF bath. Thus, during the insoluble fraction test, these oligomers may diffuse out of the system, which may account for the low cross-linking content of the cross-linked material. Thus, the gel content achieved for our samples is lower than the values obtained by Kim *et al.*^36^, who used PEO and room temperature ionic liquids (RTILs). RTILs may not take part in the chemical reaction, thus a higher insoluble content was obtained when they calculated the insoluble fraction with respect to the PEO content. Here, we propose the use of tetraglyme instead of RTIL, due to the presence of accessible protons in tetraglyme that can take part in the dehydrogenation reaction under UV irradiation in the presence of light sensitive ketyl species.

FTIR studies (Figure S3 in ESI) were performed on PTL-1 sample with and without UV curing to unravel any drastic difference induced by the irradiation. The FTIR spectra are identical and no noticeable changes are observed, which confirms that the soft cross-linking strategy does not induce any drastic modification in the polymeric components as well as with respect to the TFSI^−^ anion. Moreover, for comparison purposes, FTIR analysis of liquid TEGDME added with 10 wt. % of LiTFSI was performed and compared to the spectra of PTL-1. Clearly, the spectra of liquid electrolyte and PTL-1 are very different. In general, in an electrolyte system three kinds of ions can be present: free ions, free ions co-existing with ion pairs and aggregates^44^. PTL-1 analysed after UV-curing was almost absent with the peaks corresponding to aggregates (1236 and 1143 cm^−1^) if compared to the LiTFSI/TEGDME system that contains the same wt. % of lithium salt. As a result, we assume that the electrolyte gets enriched with free ions and neutral ion pairs, which can move faster in the polymer matrix.

### Electrochemical characterization

**Figure 4:**
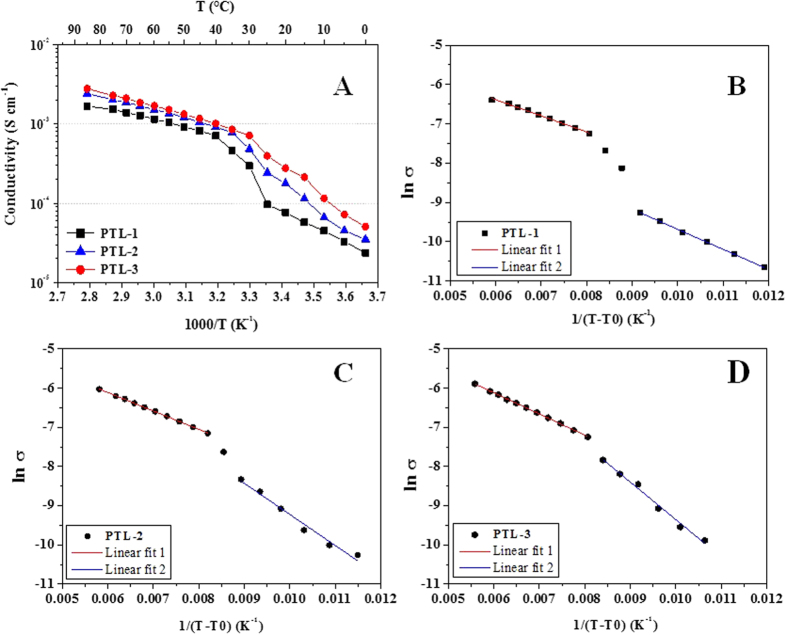
(**A**) Arrhenius plot showing the ionic conductivity *vs.* temperature for ISPEs prepared with various LiTFSI content. (**B–D**) VTF fitting of the samples PTL-1 to PTL-3.

Electrochemical impedance spectroscopy (EIS) analysis was carried out between 0 and 85 °C. The Arrhenius plots for all samples are shown in Fig. 4A. The plots of PTL-1 to PTL-3 demonstrate the influence of the lithium salt when PEO to tetraglyme ratio is 1:1. It is widely accepted that ionic conductivities exceeding 0.1 mS cm^−1^ at room temperature are necessary for an electrolyte to function in real battery configuration. Nevertheless, the crystalline domains of PEO-based polymers restrict the ionic mobility. Recently, Khurana *et al.*^45^ reported a cross-linked –EO– based polymer electrolyte showing an ionic conductivity higher than 0.1 mS cm^−1^ at 25 °C. In our work, we are able to achieve even improved ionic conductivities (0.40 mS cm^−1^ for PTL-3) for interlinked PEO-based solid polymer electrolytes. The conductivity values increase with an increase in the salt concentration, then reaching the maximum for PTL-3 where the −EO− to Li ratio is 23:1. However, the difference is not huge; indeed, all membranes demonstrate conductivity values ≥0.1 mS cm^−1^ at 25 °C. We decided not to increase the salt concentration further as for the ion pairing nature of TFSI-based salts, which strongly influences the ionic mobility, probably due to the saturation of the hopping sites^46^. It is worth mentioning that the sample retains good elastic and mechanical integrity under stress. After several days of conductivity tests, which were carried out under 10 N pressure, it was observed that the membrane retains its size and shape without any noticeable damages around the edges. Indeed, the thickness variation after the test was <2%. This is an encouraging result as ISPEs are super soft, low *T*_g_ and highly plasticized. Further, it confirms that no leakage of tetraglyme from the polymer matrix occurs.

Generally, for polymer electrolytes the dependence of ionic conductivity upon the temperature is not straightforward. The overall plot that starts from 0 to 85 °C does not exhibit a linear behaviour. Between 0 to 30 °C, the conductivity increases with Vogel–Tamman–Fulcher (VTF) dependence for all ISPEs. The same behaviour (see Fig. 4B–D) is observed above the melting point between 35 and 85 °C. The deflection around 30 °C reflects the phase transitions occurring due to the melting of crystalline regions or rearrangement of –EO– moieties, which is in agreement with the behaviour observed by DSC analysis (see Table 2).

The mechanism of ionic conductivity in polymer electrolytes can be understood from activation energy (*E*_a_) calculations. *E*_a_ was calculated by fitting the conductivity values with VTF equation^22^. The corresponding plots were used to determine the *E*_a_ of the electrolyte system. The VTF equation is believed to describe the conduction behaviour of highly concentrated liquid electrolytes and molten salts^47^. As listed in Table 2, *E*_a_ varies from 4.3 to 7.9 kJ mol^−1^, when the data are fitted with VTF equation below the deflection point, in the Arrhenius plot of Figure 4A. When the curves are fitted above the deflection point (35–85 °C, *E*_a_’), values ranging from 3.4–4.5 kJ mol^−1^ are obtained. The values obtained are superior to the data reported in the recent literature^48^. Above 0 °C, the ionic conductivity increases with a VTF-like dependence for all the samples. The discontinuities around 30 °C may be related to thermal transitions, which may include chain rearrangement, dielectric relaxations or melting of crystalline domains, formed by the PEO chains between two cross-linking points. Noteworthy, *E*_a_ increases with an increase in the salt concentration, which is ascribed to the formation of ion pairs and increased viscosity of the polymer matrix. Thus, the high ambient temperature conductivity values of the PTL series of electrolytes are predominantly associated with high ionic mobility.

The Li^+^ ion transference number (*t*_Li+_)^49^ was calculated using the method reported by Abraham *et al.*^22^ who have also considered the resistance changes occurring due to side reactions. An optimum *t*_Li+_ is necessary for the functioning of a polymer electrolyte in a Li-ion cell. Low *t*_Li+_ may induce the build-up of anion concentration gradients, which may lead to salt decomposition and precipitation. Low *t*_Li+_ may also induce dendrites growth in Li-metal cells, which is one of the major obstacles restricting the widespread intrusion of such batteries into the market 50,51,52,53. In the present system, sample PTL-1 (Figure S4) shows the highest transference number (0.55 ± 0.06, Table 2) at 25 °C. It is worth noting that at higher salt concentrations, *t*_Li+_ reduces to smaller values, which may be ascribed to the formation of anion pairs or aggregates. Overall, the transport number in the PTL series of samples is comparatively higher than the classical literature data on polymer electrolytes, but it is close to the data obtained for systems that contain tetraglyme as co-solvent^54^,^55^,^56^. The reasons for such a high number is the absence of ion pairs, or the presence of more free ions and neutral ion pairs. Moreover, the oligomerisation of tetraglyme moieties weakens the coordination between Li^+^ ions and O atom of TEGDME. This facilitates the movement of Li^+^ ions inside the polymer matrix leading to improved transference number values. It was previously demonstrated by Kriz *et al.*^55^ that tetraglyme can loosen the coordination of Li^+^ ions with –EO– units of PEO chains, resulting in improved ion mobility, and might also enable Li^+^ ions to decouple from ion pairs. If one considers that these numbers are obtained at 25 °C for a truly solid polymer electrolyte, the results are surprisingly encouraging.

The Li^+^ ion diffusion coefficient (*D*_Li^+^_) can fit very well with the previously measured ionic conductivity and transference number. *D*_Li^+^_ (see Table 2) was estimated using the method reported by Ma *et al.*^56^. Typical responses are noted as natural logarithm of potential (*V*) versus time (*t*) at 25 °C. The results are in good agreement with the corresponding *t*_Li^+^_ values. Moreover, PTL-1 shows the highest value (5.6 × 10^−6 ^cm^2^ s^−1^), which is at least one order of magnitude higher than the literature reports for similar systems^57^,^58^,^59^. The presence of free TFSI^−^ ions and neutral ion pairs, which can move faster due to the reduced solvent salt interactions, thereby increases the disorder in the polymer matrix^44^. This result is in agreement with the conductivity, transport number and diffusion coefficient studies as well as supported by FTIR studies.

**Figure 5:**
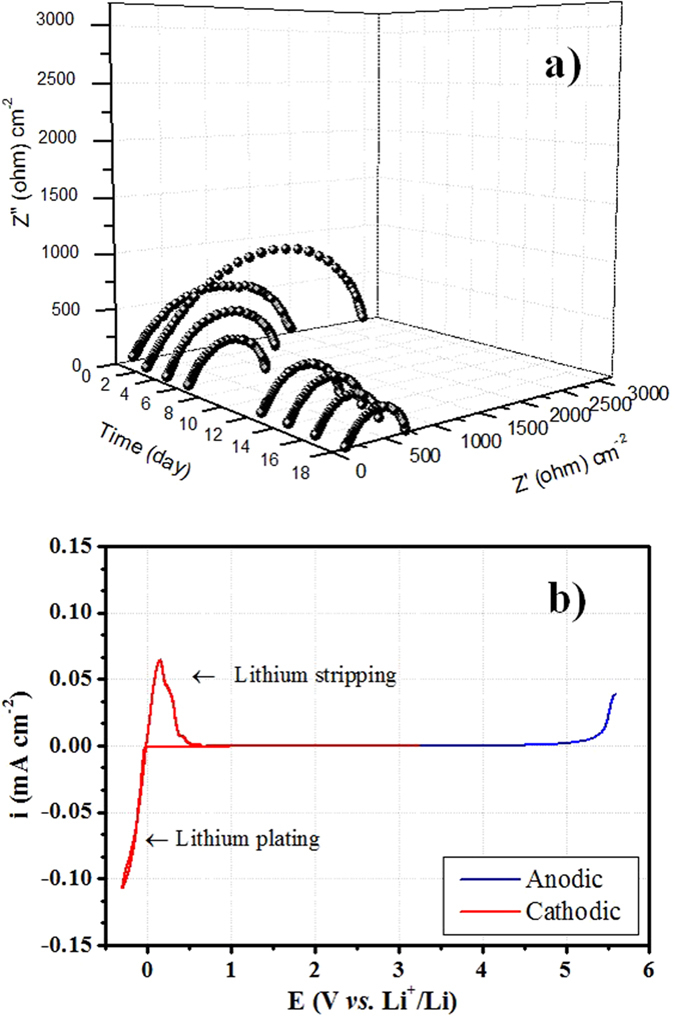
(**a**) 3D Nyquist plot representing the evolution of the interfacial resistance with time for sample PTL-1, using the Li/PTL-1/Li cell configuration. (**b**) Electrochemical stability window (anodic and cathodic scan) of PTL-1. The tests were performed at 25 °C.

A deep understanding of the interfacial properties between the lithium metal electrode and the polymer electrolyte is necessary in order to provide more insight over the factors controlling the recharge ability of lithium-based polymer batteries. The PTL-1 sample was examined in terms of compatibility (interfacial stability) with the lithium metal electrode. Sample PTL-1 was selected for further characterizations due to the optimal characteristics exhibited during the previously discussed analyses. As shown in Fig. 5a, the PTL-1 based lithium symmetric cell shows stable resistance after few days of testing. Indeed, the resistance increases during the initial days of storage indicating an appropriate formation of a thin solid electrolyte interface (SEI) layer at the surface of the lithium metal electrode^60^. The resistance rapidly decreases and stabilizes at around 700 Ω cm^−2^ after about 6 days, accounting for the very stable interfacial characteristics of the sample. This behaviour is typical of most of the polymer electrolytes, and is clearly related to the initial formation of the SEI layer, its stabilisation and, then, the improved contact achieved with time at the interface between the lithium metal electrode and the polymer electrolyte^61^,^62^.

Electrochemical stability window (ESW) is a fundamental parameter that determines the durability and energy output of a lithium cell. Figure 5b shows the electrochemical stability of PTL-1 sample towards anodic oxidation and cathodic reduction reactions. The test was performed at 25 °C. From the cathodic profile, the almost ideally reversible lithium plating and stripping processes are well evidenced. Overall, a wide electrochemical window is accessible for the electrolyte to be safely used between 0 to above 5.2 V *vs.* Li/Li^+^. Such a high anodic stability window can be explained by the simultaneous oxidative decomposition of both the TFSI^−^ anions, tetraglyme moieties and PEO matrix in the high potential regions^58^. In particular, the presence of tetraglyme in the electrolyte matrix increases the overall oxidation stability^54^. Even though the CH_2_-CH_2_-O- chemical moiety is same for both PEO and tetraglyme, the difference may arise from the –CH_3_ end group of tetraglyme, which avoids the interaction between electrode surface and the –OH terminal groups of PEO. However, the oxidation stability is anyway superior than the pure PEO-based system, and this is an intriguing aspect of this electrolyte. This value is excellent when one envisages the application with high voltage cathode materials.

**Figure 6:**
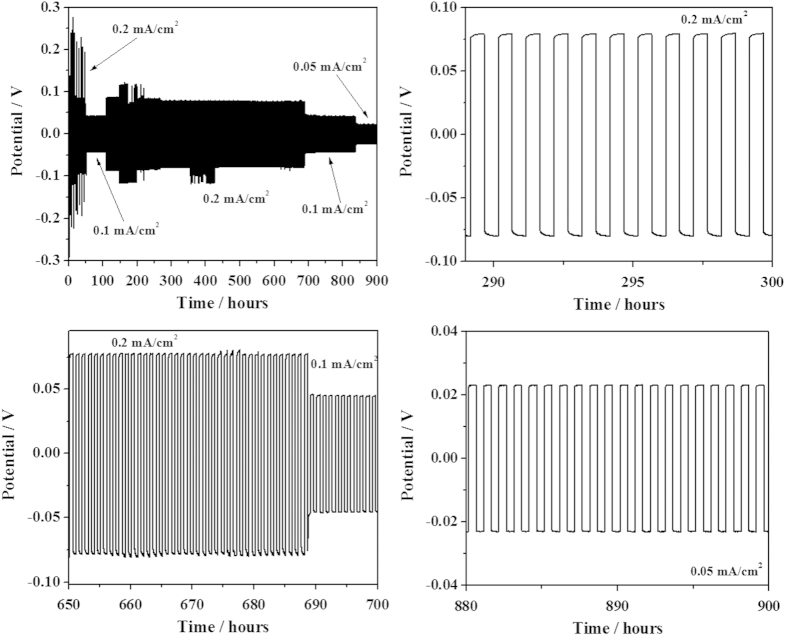
Potential *vs.* test time of lithium stripping and plating of a symmetrical lithium cell at various current rates (i.e., 0.05, 0.1, 0.2 mA cm^−2^) at 20 °C.

Many researchers developed polymer electrolytes that exhibited ionic conductivity values as high as 0.1 mS cm^−1^ at 25 °C^57^,^63^, but very few investigated the lithium dendrite nucleation and growth resistance in real cell configuration^15^. Inspired by the dendrite studies reported by Balsara *et al.*^64^ and Khurana *et al.*^45^ we performed galvanostatic lithium plating/stripping measurements in symmetric Li/PTL-1/Li cells to determine the lifetime of our lithium metal polymer batteries. Such a test is of utmost importance when very long-term ageing of lithium metal polymer cells is envisaged^50^. Measurements were performed at 0.1 and 0.3 mA cm^−2^ current densities at 25 °C (3 h Li-plating and 3 h Li-stripping). When the current density is increased from 0.1–0.3 mA cm^−2^, a large change in potential is observed due to the formation of a dendritic short circuit 50. The cycling results of PTL-1 at 0.1 and 0.3 mA cm^−2^ current density are shown in Figure S5. Indeed, rather than the total charge deposited within the time, the current rate of 0.3 mA cm^−2^ induced an over potential as demonstrated in Figure S5. Thus, ISPE can be safely used at 0.1 mA cm^−2^, which is assumed as a remarkable value for a solid electrolyte system operating at ambient temperature. Prolonged galvanostatic cycling tests were performed (Fig. 6) at 0.1 and 0.2 mA cm^−2^ with plating and/or stripping steps lasting for 30 min. This test assures the durability and safe operation of the ISPEs in lithium metal cells conceived for ambient temperature applications. The total charge carried during the plating / stripping process is not very high, however, one can hypothesise that this is a good indication towards pursuing this path for future studies.

**Figure 7:**
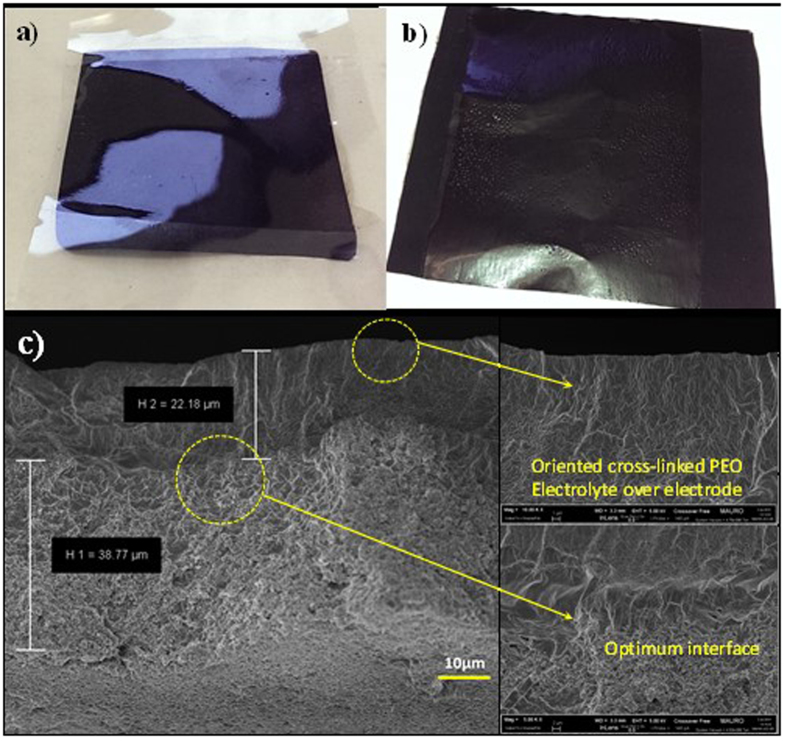
(**a)** Photograph of bare TiO_2_ based electrode and electrolyte before UV curing. (**b)** Freshly prepared self-supporting multiphase electrode/electrolyte composite obtained by direct hot-pressing and *in situ* photopolymerisation of the polymer electrolyte over the TiO_2_-electrode film supported over copper foil. (**c)** Cross-sectional FESEM images showing the optimum interface achieved after UV curing.

To demonstrate its practical application, PTL-1 was assembled in a lab-scale all-solid-state Li-polymer cell, and galvanostatically cycled at ambient temperature. The cell was assembled by combining a Li-metal anode with an electrode/electrolyte composite prepared by *in situ* UV cross-linking the ISPE directly over the TiO_2_-based working electrode (details in experimental). One of the major drawbacks of typical truly solid polymer electrolytes is the insufficient contact between the electrode active materials and the polymer matrix. Thus, the direct cross-linking step over the electrode surface is fundamental to obtain a good electrode/electrolyte interfacial adhesion. The process enables us to obtain a stable and thin (≈30 μm) polymer electrolyte film with uniform distribution over the electrode. The general aspects of the bare electrode, electrolyte and the final aspect of the TiO_2_-based electrode film are shown in Fig. 7 (a,b). The cross-sectional FESEM images (Fig. 7c) show an intimate contact achieved between the active materials and the polymer electrolyte. Particularly at higher magnifications, it can be clearly observed that the electrolyte layer creates a conformal coating by following the contours of the electrode particles. This leads to improved active materials utilisation at the interface between the electrode and the polymer electrolyte, which correspondingly improves the specific energy and power of the cell. The oriented cross-linked polymer electrolyte morphology is observable on top of the electrode (Fig. 7c) along with the optimum interface and interpenetration between the electrode active material particles and the electrolyte.

**Figure 8:**
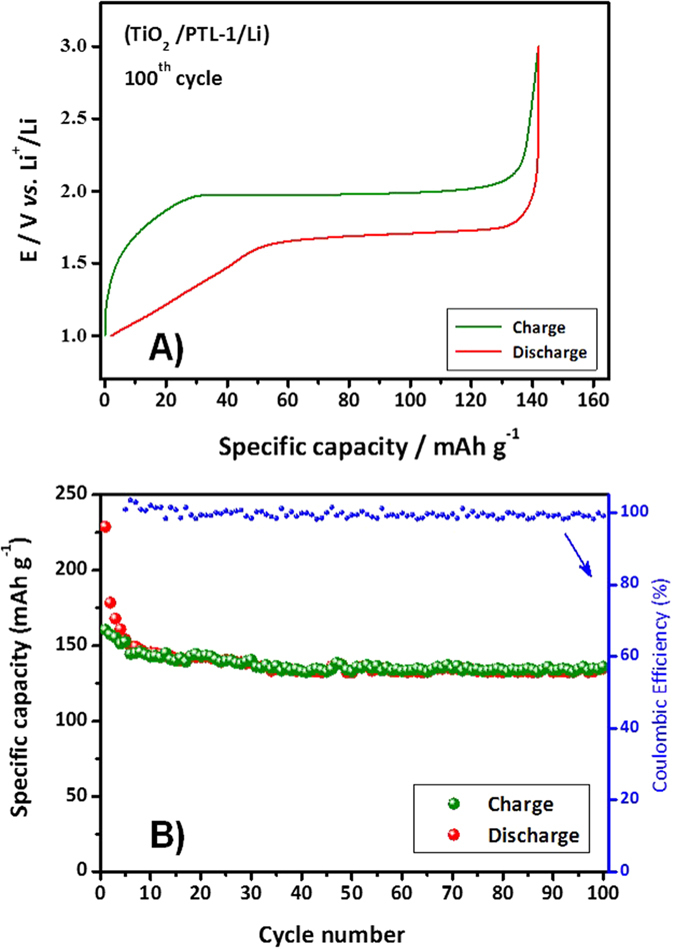
(**A**) Representative charge/discharge profiles of a cell assembled with the configuration of Li/PTL-1/TiO_2_. The cycling test was performed at 20 °C at a current density of 0.1 mA cm^−2^. (**B)** Graph illustrating the specific capacity *vs.* number of cycles along with Coulombic efficiency.

The response of the cell at 0.1 mA cm^−2^ is shown in Fig. 8 in terms of galvanostatic charge/discharge profiles and specific capacity *vs.* cycle number. The cell was prepared by just contacting a lithium metal foil at the polymer side of the electrode/electrolyte composite. The constant current charge/discharge profiles shown in plot (A) reflect the good properties of the electrolyte system, showing rather flat potential plateaus during charge and discharge cycles. These are typical of the biphasic Li^+^ extraction/insertion mechanism of crystalline TiO_2_ anatase, with a steep potential increase/decay at its end. The polarization is rather limited, which accounts for an efficient redox reaction kinetics, due to the limited internal resistance at the electrode/electrolyte interface as well as the limited cell over potential contributions. In general, the material shows good cycling stability, as for the good overlapping of the charge/discharge curves, accounting for a Coulombic efficiency close to 100%. Although the specific capacity obtained is lower if compared to the same anatase electrode material at same current density in liquid electrolyte, the polymer cell shows good capacity retention approaching 90%. This is a convincing indication of the good interfacial contact between the electrodes and the electrolyte separator. Noteworthy, the cycling response of the all-solid polymer cell is clearly at the level of the corresponding TiO_2_/Li cell assembled with a liquid electrolyte made of 1M LiTFSI solution in tetraglyme (TEGDME) (Figure S6), which accounts for the almost full capacity delivery when using the all-solid-state configuration.

The PTL-1 also demonstrated the ability to be galvanostatically cycled at 0 and 25 °C in lab-scale Li cell comprising a LiFePO_4_ cathode. Proof-of-concept charge/discharge profiles are shown in Figure S7, which clearly enlighten the possibility of designing an all-solid polymer battery system that functions at low temperature even with various electrode materials. The approach can be extended to other energy-related device applications like Na-ion batteries, dye-sensitized solar cells^65^ and supercapacitors, owing to its simple, scalable, economic and eco-friendly preparation method, and a great potential to serve as a light-designed cell component.

## Conclusions

The super soft polymer electrolyte network was architectured from a thermoplastic polymer matrix of known molecular weight using the rapid and cost-effective *in situ* photopolymerisation technique. A multidisciplinary approach was adopted to understand the role of photopolymerisation in tailor making the integral and requisite properties of the resulting polymer electrolyte to achieve acceptable conductivity, ionic mobility and resilience towards dendrite-induced short circuit reactions. Significantly, the feasibility of using such novel electrolyte in real cell configuration at ambient temperature with various nanostructured electrodes was established by suitably adopting *in situ* polymerization directly over the electrode films. The obstacles related to hazardous dendrites and reactivity towards Li-metal were nullified, leading to the assembly of superior Li-ion and Li-metal cells conceived for applications that demand high energy and/or power, including smart-grid storage and electric-/hybrid-electric vehicles. We anticipate that the proposed approach would lead to a rational designing to address the significant challenges of Li-ion polymer batteries.

## Experimental Section

### Materials and Methods

The reactive formulations were based on poly(ethylene oxide) (PEO, average *M*_w_ 100,000, Sigma-Aldrich), and an active plasticiser bis[2-(2-methoxyethoxy) ethyl]ether (tetraglyme, TEGDME, Sigma-Aldrich) along with bis(trifluoromethane) sulfonimide lithium salt (CF_3_SO_2_NLi-SO_2_CF_3_, LiTFSI, battery grade, Solvionic) as Li^+^ ions source. The photo-induced hydrogen abstraction facilitator was 4-methylbenzophenone (MBP, Sigma-Aldrich).

The preparation of interlinked polymer electrolyte network did not involve any solvents or long/tiring steps. The materials were grinded in appropriate proportions at 70 °C. After blending, the resulting mixture was hot-pressed at 90 °C for 15 min to obtain a homogeneous thin film. The film was later exposed to UV light for 6 min to reticulate the reactive species, thus obtaining the cross-linked ISPE of average thickness 90 ± 10 μm. The solid and non-tacky film was peeled off from the substrate (Mylar sheet) and used for further characterizations. The procedure was carried out in a dry room (10 m^2^, R.H. <2% ± 1 at 20 °C) produced by Soimar (Caluso, Italy). As the prepared membrane contains all the necessary components for a functional electrolyte, no additional steps were required for further characterization.

### Characterization of the interlinked solid polymer electrolytes

The insoluble fraction (gel content) was determined as follows: samples of known weight were kept in a stainless steel metal net, and subsequently extracted with acetonitrile (CH_3_CN, ACN, Sigma-Aldrich) to dissolve the non-cross-linked polymer chain components^66^. Extraction included 18 h of residence time for the solvents to remove soluble contents from the membrane at 25 °C. Instead of using CHCl_3_, ACN was used due to the solubility of the components used for membrane preparation. Moreover, CHCl_3_ is suspected for carcinogenic activity. The cross-linked (insoluble) fraction was then calculated by dividing the mass of the dry sample left over after the extraction by the mass of the original sample (relative error = ±1%).

The overall morphology was investigated by field emission scanning electron microscopy (FESEM) on a ZEISS Supra 40, equipped with an energy dispersive X-ray spectrometer (EDX). For cross-sectional morphology characterization, the samples were cracked after dipping in liquid nitrogen to avoid any change in the morphology. Then, the samples were subjected to metallization by sputtering a very thin Cr layer (~10 nm) to minimize the effect of the electron beam irradiation.

The glass transition temperature (*T*_g_) of the samples was evaluated by differential scanning calorimetry (DSC) with a METTLER DSC-30 (Greifensee, Switzerland) instrument. In a typical measurement, the electrolyte samples were cooled from ambient temperature to –85 °C and then heated at 5 °C min^−1^ up to 120 °C. The *T*_g_ was calculated as the midpoint of the heat capacity change observed in the DSC trace during the transition from glassy to rubbery state. The thermal stability was tested by thermo-gravimetric analysis (TGA) using a TGA/SDTA-851 instrument from METTLER (Switzerland) over the temperature range between 25 and 600 °C under N_2_ flux at a heating rate of 10 °C min^−1^.

The mechanical properties were determined by dynamic-mechanical thermal analysis (DMTA) on a MK III Rheometrics Scientific Instrument at 1.0 Hz frequency on tensile configuration at a heating rate of 5 °C min^−1^. The specimen size was 20 mm × 4 mm × 0.2 mm. The storage modulus and the loss factor were measured from −80 to 30 °C. The Fourier Transform Infrared (FT-IR, NICOLET-5700) spectroscopy (400–4000 cm^−1^, resolution 2 cm^−1^) was carried out at 25 °C, on extremely thin films sandwiched between gold plates with an orifice. Tensile tests were carried out according to ASTM Standard D638, using a Sintech 10/D instrument equipped with an electromechanical extensometer (clip gauge).

Gel permeation chromatography (GPC) was performed to examine the effect of UV curing using an Agilent Technologies 1200 Series (USA) instrument. The instrument was equipped with a refractive index (RI) detector and two Waters Styragel columns (HT2 and HT4) conditioned at 35 °C. A sample of cross-linked polymer was weighted, transferred into a Whatman glass microfiber thimble and extracted with THF for 24 hours using a Soxhlet apparatus. After extraction, a small quantity of the soluble fraction was transferred to a vial and left open to evaporate THF. The obtained solid was then diluted with chromatography-grade THF and used for GPC measurement.

### Electrochemical characterization methods

The following procedures were performed in the inert atmosphere of a dry glove box (MBraun Labstar, O_2_ and H_2_O content <1 ppm) filled with extra pure Ar 6.0. The ionic conductivity was determined by electrochemical impedance spectroscopy (EIS) analysis of cells assembled by sandwiching the ISPE between two stainless steel (SS-316) blocking electrodes (area 2.54 cm^2^). A PARSTAT-2273 potentiostat/galvanostat/F.R.A. (Frequency Response Analyzer) by Princeton Applied Research (USA) was used for measurements at various temperatures between 100 kHz and 1 Hz at the open circuit potential (OCV). The bulk resistance of the samples was calculated from the impedance curve. Then, the ionic conductivity was calculated based on the following equation:


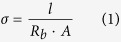


where *σ* is the ionic conductivity (S cm^−1^), *R*_b_ the bulk resistance, *l* and *A* are the thickness and the area of the specimen, respectively. The tests were performed using a climatic chamber (UFE-400 Memmert GmbH, Germany). The bulk resistance was given by the high frequency intercept determined by analysing the impedance response using a fitting program provided by Electrochemistry Power Suite software (V 2.58). Samples were kept at 80 °C overnight and then tested from 0 to 85 °C at every 10 °C. Measurements were repeated at least three times.

ISPEs were tested for compatibility (interfacial stability) with the Li-metal electrode by monitoring the evolution of the impedance response of a symmetrical non-blocking Li/ISPE/Li cell with time at 25 °C under OCV condition.

The Li-ion transference number (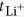) was measured^18^,^39^,^54^ at 25 °C by combined AC impedance and DC polarization measurements of a Li/ISPE/Li cell, as explained by Watanabe^67^ and Bruce^49^. The cell was kept at 80 °C overnight to achieve an intimate contact, and a stable interface between the electrolyte and the electrodes. Successively, after retaining at 25 °C, a DC potential (ΔV = 10 mV) was applied until a steady current was obtained (generally 3–5 hours), and the initial (*I*_0_) and steady (*I*_ss_) currents that flow through the cell were measured. Simultaneously, impedance spectra were recorded (100 KHz and 0.1 Hz), with an oscillating potential of 10 mV, before and after DC polarization. Subsequently, the initial (*R*_0_) and final (*R*_ss_) bulk resistances of the electrolyte, and the initial (*RC*_0_) and final (*RC*_ss_) charge transfer resistances (Ω) of the interfacial layers of the Li metal electrode/electrolyte were derived. Using these measured values, 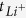 was calculated by following equation:


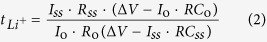


The electrochemical stability window (ESW) of the ISPEs was evaluated at 25 °C by linear sweep voltammetry (LSV) in two electrodes cells using a CHI-660 electrochemical workstation. Separate LSV tests were carried out on each of the samples to determine the cathodic and anodic breakdown voltages. Cell configuration for anodic scan (OCV to 6 V *vs.* Li/Li^+^): SS-316 as the working electrode, Li-metal discs as both the counter and the reference electrodes, and ISPE as the electrolyte (area 2.54 cm^2^)^68^. Cell configuration for cathodic scan (OCV to −0.2 V *vs.* Li/Li^+^): Cu disc as the working electrode, Li metal discs as the counter and the reference electrodes, ISPE as the electrolyte at a scan rate of 0.100 mV s^−1^. The current onset of the cell was associated with the decomposition potential of the electrolyte.

The activation energy was calculated from conductivity values obtained at various temperature and the resulting values are fitted with Vogel–Tamman–Fulcher (VTF) equation, which is typically used to describe the relation between viscosity and temperature near the *T*_g_ of the polymer matrix. The equation used is given below:,


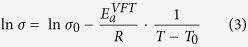


where *σ* is the ionic conductivity, *E*_a_^VFT^ is equivalent to the activation energy, *R* is the gas constant, *T* is the experimental temperature and *T*_0_ is the temperature which is 50 °C below the *T*_g_.

The salt diffusion coefficient of the ISPEs was estimated using the method proposed by Ma *et al.*^56^. In this case, the cells are polarized at 5 mV before the potential is interrupted. Once the potential is interrupted, the cell is kept at OCV until a stable state is achieved. Later, the profiles are plotted as the natural logarithm of potential (V) versus time (t). The *D*_Li+_ values were calculated from the slope of the linear curves using the following equation:


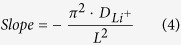


where *L* is the thickness of the ISPE under study.

Galvanostatic charge/discharge cycling tests were performed on Li metal cells of area 2.54 cm^2^ using both TiO_2_ (potential scan range 1–3 V *vs.* Li/Li^+^) and LiFePO_4_ (potential scan range 2.5–4 V *vs.* Li/Li^+^) based working electrodes with an ARBIN BT2000 battery tester. The working electrodes were prepared from a slurry that contains TiO_2_ (Hombicat-100) or LiFePO_4_ (Clariant-LP2), carbon black and PVdF in 70:20:10 weight ratio, respectively. The slurry was deposited over Cu (or Al) foil and later dried overnight (120 °C). The typical preparation for the electrode-electrolyte composites followed the procedure reported elsewhere^69^. In a typical preparation procedure, appropriate amounts of PEO, tetraglyme, LiTFSI and MBP were mixed at 70 °C and mechanically grinded to obtain a viscous paste-like mixture. This mixture was later deposited over a composite electrode film, and hot pressed (20 bar, 90 °C) for 15 min to obtain a uniform coating over the electrode surface. This setup was exposed to UV light for 6 min to obtain a cross-linked polymer electrolyte system. Then, electrolyte/electrode disks (area 2.54 cm^2^) were cut from the sheet and dried under vacuum overnight at 40 °C prior to cell assembly. The electrode films used in this study were pressed prior to pre-polymer deposistion, which can retain the some pores/voids for the electrolyte components to accommodate while overall processing. The electrodes we used in the present case are not pressed/calendared.

## Additional Information

**How to cite this article**: Porcarelli, L. *et al.* Super Soft All-Ethylene Oxide Polymer Electrolyte for Safe All-Solid Lithium Batteries. *Sci. Rep.* **6**, 19892; doi: 10.1038/srep19892 (2016).

## Supplementary Material

Supplementary Information

## Figures and Tables

**Table 1:** Composition of ISPEs, along with their *T*_g_ and gel-content values.

	LiTFSI^ψ^	PEO^ψ^	TEGDME^ψ^	*T*_g_^§^	Gel^ψ^	EO:Li
PTL-1	10	41.3	41.2	−34	42 ± 2	54:1
PTL-2	15	38.8	38.7	−38	39 ± 2	35:1
PTL-3	20	36.3	36.2	−44	37 ± 3	23:1

MBP content is 7.5 wt. % of the total weight of materials. ^Ψ^units in wt. %. ^§^unit in °C.

**Table 2:** Ionic conductivity (*σ*, at 25 °C) and related characteristics of ISPEs prepared with different salt content.

Name	*E*_a_/*E*_a_′ ^#^	*t*_Li_^+^	D_Li+_^$^	σ*
**PTL-1**	4.3**/**3.4	0.55 ± 0.06	5.6 × 10^−6^	0.11
**PTL-2**	6.6**/**3.9	0.48 ± 0.02	1.2 × 10^−7^	0.24
**PTL-3**	7.9**/**4.5	0.32 ± 0.08	2.1 × 10^−8^	0.40

*E*_a_ is the activation energy before deflection (0 to 30 °C). *E*_a_′ is the activation energy after deflection (35 to 85 °C). *t*_Li+_ is the lithium transference number; *D*_Li+_ is the lithium diffusion coefficient. *mS cm^−1^. ^#^kJ mol^−1^. ^$^cm^2^ s^−1^.
