# Supplementary material for: Super Soft All-Ethylene Oxide Polymer Electrolyte for Safe All-Solid Lithium Batteries
Source: Sci Rep. 2016 Jan 21;6:19892. doi: 10.1038/srep19892 (PMC4726218; doi:10.1038/srep19892)
Supplement: Supplementary Information [file srep19892-s1.doc]

**Super Soft All-Ethylene Oxide Polymer Electrolyte for Safe All-Solid Lithium Batteries**

*Luca Porcarelli, Claudio Gerbaldi*, Federico Bella, and Jijeesh Ravi Nair**

**Keyword:** polymer electrolyte, photopolymerization, in situ crosslinking, lithium battery

**A thermoplastic polymer matrix sufficiently cross-linked, mechanically durable super soft electrolyte membrane is prepared using a rapid and cost-effective *in situ* photo(co)-polymerization technique.** Impressive ambient temperature ionic conductivity values are obtained, along with wide electrochemical stability window, excellent lithium transference number and stable interfacial stability upon prolonged contact with the lithium metal electrode.

**
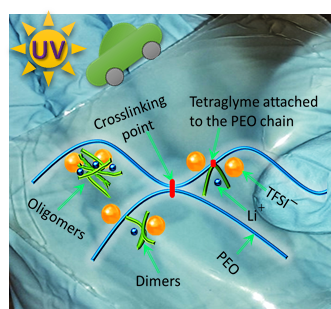
**

**Supporting Information**

**Super Soft All-Ethylene Oxide Polymer Electrolyte for Safe All-Solid Lithium Batteries**

*Luca Porcarelli, Claudio Gerbaldi*, Federico Bella, and Jijeesh Ravi Nair**

**A**

**B**

**C**

**Figure S1. A,B)** DMTA profiles of PTL-1 sample, in which the results are shown for tests carried out between ‒80 °C to 30 °C. Above 20 °C, the test failed due to the extra-soft characteristics of the polymer membrane if compared to the applied force from the instrument (10 N); **C)** Tensile tests carried out on PTL-1 sample according to ASTM Standard D638, using a Sintech 10/D instrument equipped with an electromechanical extensometer (clip gauge) are reported as Stress/Strain curve.


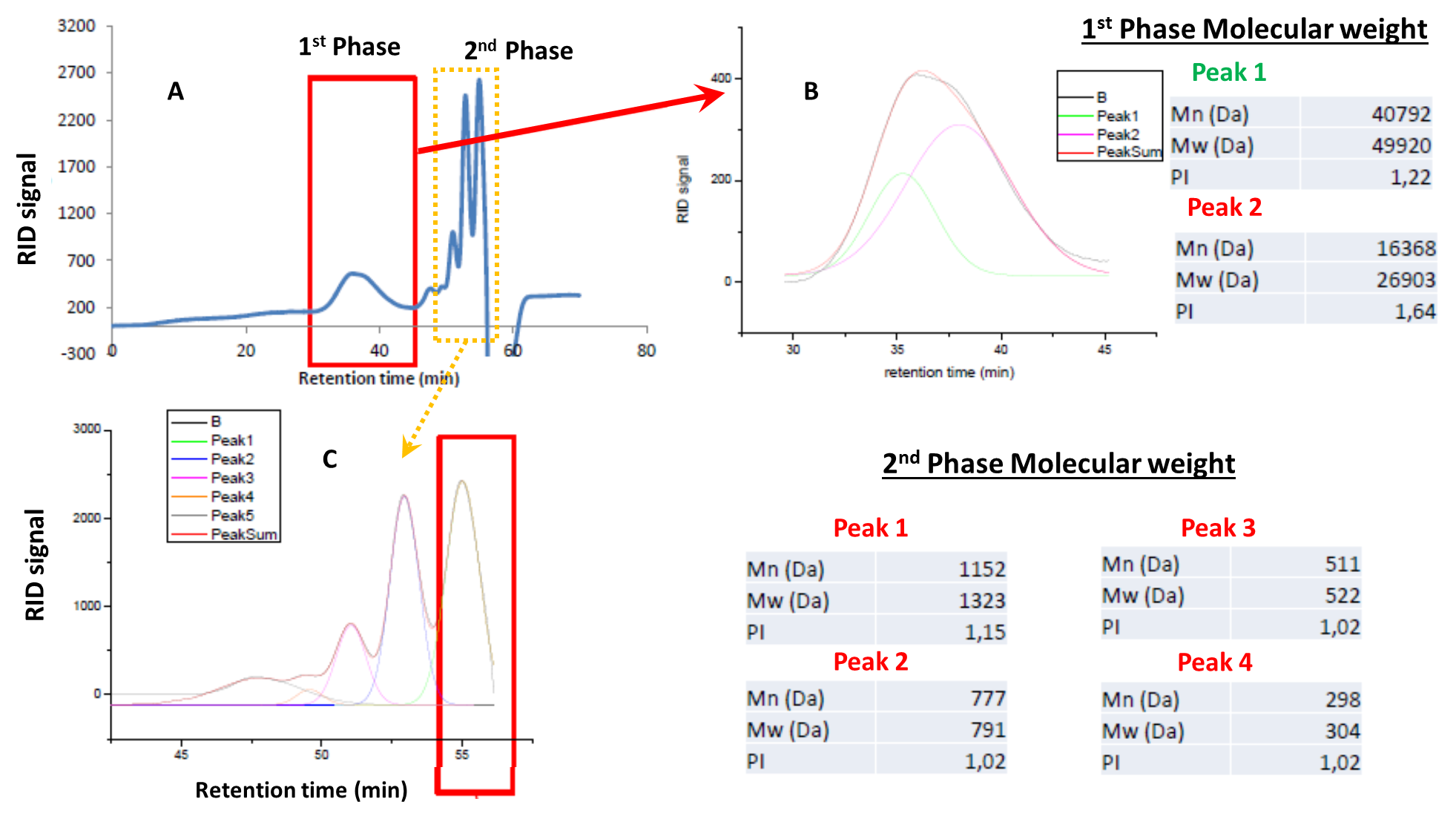


**Figure S2.** Gel permeation chromatography (GPC) was performed to examine the effect of UV curing on tetraglyme molecules. The GPC graphs of PTL-1 polymer are shown along with the molecular weight and polydispersity index.

**Figure S3. A)** FTIR profiles of PTL-1 before and after UV curing; **B)** FTIR profiles of PTL-1 and liquid TEGDME-LiTFSI. The tests were carried out at room temperature.


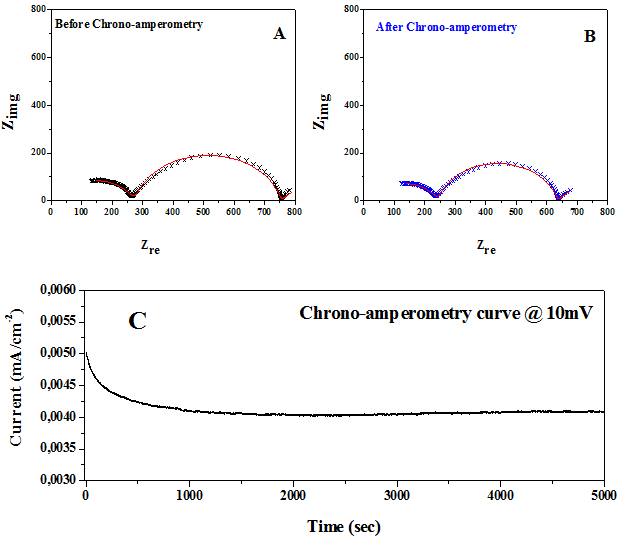


**Figure S4. A-B)** Nyquist plot of a membrane assembled in a symmetric cell (Li/PTL-1/Li) and tested at 20 °C in the frequency range between 2 MHz and 0.1 Hz, before and after the chrono-amperometry test; **C)** Chrono-amperometry curves obtained for PTL-1 containing cell, tested at 10 mV polarization until obtaining a steady state current under open circuit potential conditions. Tests were carried out for all the samples, but PTL-1 is showed as the representative. The values for all the samples are tabulated in Table 1 in the manuscript.

**Figure S5.** Galvanostatic cycling curve obtained for Li/PTL-1/Li symmetric cell at a fixed current density of 0.1 mA cm^−2^ at 20 °C. The cell showed good plating and stripping of lithium on the electrodes at 0.1 mA cm^−2^, but when the current was increased to 0.3 mA cm^−2^, the cell was rapidly short circuited indicating that the maximum current density that can be applied at room temperature is lower than 0.3 mA cm^−2^. If one consider the application at room temperature, this is a very high value.

**Figure S6.** Charge/discharge curve of lithium test cell assembled with a configuration of Li/PTL-1/TiO_2_ using the liquid electrolyte solution made of 1M LiTFSI in tetraglyme (TEGDME). The cycling test was performed at 20 °C at current density of 0.1 mA cm^–2^ to be comparable to the solid PTL-1-based cell.


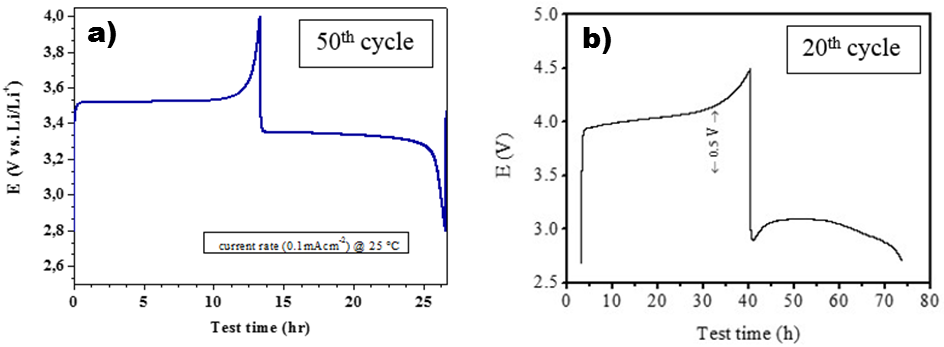


**Figure S7.** Charge/discharge curve of lithium test cells assembled with a configuration of Li/PTL-1/LiFePO_4_. The cycling tests were performed at 25 °C (a) and 0 °C (b) at current densities of 0.1 and 0.01 mA cm^–2^, respectively. ///At 0 °C, the all-solid-state cell showed an increased polarization, but still maintaining an appropriate functioning and demonstrated typical charge/discharge plateaus of LiFePO_4_ electrode. The charge/discharge profiles depicted in the figures are extracted from prolonged cycling tests, and then just normalized to test time.

**C**
